# Callous–unemotional traits in adolescents with autism spectrum disorder

**DOI:** 10.1192/bjp.bp.114.159863

**Published:** 2015-11

**Authors:** Virginia Carter Leno, Tony Charman, Andrew Pickles, Catherine R. G. Jones, Gillian Baird, Francesca Happé, Emily Simonoff

**Affiliations:** **Virginia Carter Leno**, BSc, MSc, Department of Child and Adolescent Psychiatry, King's College London, Institute of Psychiatry, Psychology & Neuroscience, London; **Tony Charman**, PhD, Department of Psychology, King's College London, Institute of Psychiatry, Psychology & Neuroscience, London; **Andrew Pickles**, PhD, Department of Biostatistics, King's College London, Institute of Psychiatry, Psychology & Neuroscience, London; **Catherine R. G. Jones**, PhD, School of Psychology, Cardiff University, Cardiff; **Gillian Baird**, FRCPCH, Guy's & St Thomas' NHS Foundation Trust, Newcomen Centre, London; **Francesca Happé**, PhD, MRC SDGP Centre, King's College London, Institute of Psychiatry, Psychology & Neuroscience, London; **Emily Simonoff**, MD, FRCPsych, Department of Child and Adolescent Psychiatry, King's College London, Institute of Psychiatry, Psychology & Neuroscience and NIHR Biomedical Research Centre for Mental Health, London, UK

## Abstract

**Background**

People with callous–unemotional traits and also those with autism spectrum disorder (ASD) display sociocognitive difficulties. However, the frequency and neurocognitive correlates of callous–unemotional traits within individuals with ASD are unknown.

**Aims**

To determine the prevalence of callous–unemotional traits in individuals with ASD and test their association with behavioural and cognitive measures.

**Method**

Parents of 92 adolescents with ASD completed the Antisocial Processes Screening Device (APSD) for callous–unemotional traits. Adolescents participated in tasks of emotion recognition, theory of mind and cognitive flexibility.

**Results**

In total 51% (n = 47) scored above a cut-off expected to identify the top 6% on the APSD. Of these 17% (n = 8) had concurrent conduct problems. Regression analyses found callous–unemotional traits were associated with specific impairment in fear recognition but not with theory of mind or cognitive flexibility.

**Conclusions**

Adolescents with ASD show high rates of callous–unemotional traits but, unlike in the general population, these are not strongly associated with conduct problems. The relationship of callous–unemotional traits to impairments in fear recognition suggests similar affective difficulties as in individuals with callous–unemotional traits without ASD.

Autism spectrum disorder (ASD) is a neurodevelopmental disorder characterised by a lack of social reciprocity and qualitative impairment in communication, and restricted, repetitive behaviours and interests.^1^ Cognitive impairments have been hypothesised to underlie the surface features of behaviour. Individuals with ASD have difficulty recognising emotions^2^ (although see Jones *et al*^3^) and understanding the mental states of others, also known as theory of mind,^4^ both of which are thought to contribute to problems in social functioning. Executive dysfunction is also reported in individuals with ASD, and may underlie restrictive and repetitive symptoms. Impairments occur on tasks of planning, response selection/monitoring and inhibition of pre-potent response in children^5^ and adolescents,^6^ as well as reduced cognitive flexibility.^7^ With regard to common coexisting behavioural problems, individuals with ASD are at higher risk of co-occurring psychopathology.^8–10^ Individuals with ASD display increased antisocial and aggressive behaviour as children^11^ and oppositional defiant disorder as adolescents.^10^ Children presenting with both ASD and oppositional defiant disorder have a more severe pattern of psychiatric symptoms than those without oppositional defiant disorder, with increased symptoms of generalised anxiety, mania, major depressive disorder and perseverative behaviours, in addition to more conduct problems.^12^

Within the heterogeneous presentation of conduct disorder in individuals without ASD, a distinct subgroup with a more severe and stable pattern of antisocial behaviour has been designated as having callous–unemotional traits.^13^ This subgroup has a distinct affective and interpersonal style, including a lack of empathy, an absence of remorse and a tendency to use others for personal gain.^13^ Callous–unemotional traits in youth are a juvenile precursor of, and conceptually similar to, psychopathy.^13^ Underlying cognitive and affective processing difficulties have been proposed to underlie atypical social functioning in individuals with callous–unemotional traits. Adolescents with psychopathic traits^14^ and children with high callous–unemotional trait scores^15^ have difficulties in the recognition of fearful faces. This inability to recognise interpersonal signals of distress has been postulated to underlie atypical empathic development.^16^ Robust and well-replicated associations between psychopathy and executive dysfunction are also reported,^17^ including difficulties with cognitive flexibility, attentional switching^18^ and response reversal.^19^ The explanatory role of attention–deficit hyperactivity disorder (ADHD) symptoms in the association between executive functioning and callous–unemotional traits is not well understood; controlling for ADHD has been reported to eradicate the relationship between executive functioning and delinquency in adolescents.^20^ Individuals with ASD and those with callous–unemotional traits can sometimes present similarly, at least on a superficial level, yet this may be as a result of different aetiological processes. Despite phenotypic similarity, twin studies report largely independent genetic influences (an overlap of 0.43) on autistic and psychopathic traits.^21^ Comparative studies have suggested that callous–unemotional traits are associated with a selective impairment in affective domains (for example the ability to care about another's feelings), whereas cognitive domains (for example the ability to understand what another is thinking; theory of mind) remain unaffected.^22–24^ Conversely, individuals with ASD appear to have difficulties in cognitive, but not affective, aspects of interpersonal tasks.^4,22–24^ Recent neuroimaging studies comparing individuals with callous–unemotional traits and those with ASD have supported the behavioural findings of distinct patterns of theory of mind and affective empathy of the two groups.^25^

Very few studies have examined the profile associated with callous–unemotional traits within individuals with ASD. Rogers *et al* reported that children with ASD and callous–unemotional traits required a longer latency to recognise sad faces, but were not impaired in intelligence, response inhibition or cognitive flexibility.^26^ Comparison of individuals with ASD who were also offenders *v.* non-offenders showed a specific impairment in fear recognition, yet unimpaired theory of mind and executive functioning in the offending group.^27^ These studies suggest that some individuals with ASD also show callous–unemotional traits, perhaps as a result of a ‘double-hit’, with the emotional processes implicated in callous–unemotional being affected, in addition to the social cognitive difficulties typically present in individuals with ASD (for example theory of mind impairment). However, whether these findings hold in larger, population-based samples remains unexplored. Individuals with this ‘double-hit’ of ASD and callous–unemotional traits may be a complex yet relatively common presentation within clinics caring for young people with ASD and additional mental health problems, and thus require further study. Previous research on callous–unemotional traits in people with ASD has used selected populations. This paper extends previous findings by addressing the behavioural and cognitive correlates of callous–unemotional traits within a population-based sample of adolescents with ASD. We first investigate the frequency of callous–unemotional traits in adolescents with ASD. We test whether the strong association between these traits and conduct problems reported in the general population also applies to those with ASD, and describe additional psychiatric symptoms associated with callous–unemotional traits. Finally, we test whether a specific impairment in fear recognition is associated with callous–unemotional traits, as in non-ASD populations, and explore whether these traits are associated with difficulties in theory of mind and cognitive functioning.

## Method

### Participants

A total of 92 adolescents with ASD, who were verbal and had an IQ ≥50 had been assessed on the relevant measures as part of the Special Needs and Autism Project (SNAP) cohort. This cohort was drawn from 56 946 children living in the South Thames area of the UK and born between July 1990 and December 1991, initially as part of an autism prevalence study (see Baird *et al*^28^ for further details). The cohort was assessed at age 12 and 16 years. Assessment at 16 years focused on the cognitive phenotype of ASD and only those who had estimated IQs ≥50 at 12 years were included.^29^ All received a consensus clinical ICD-10^30^ ASD diagnoses made using the Autism Diagnostic Interview-Revised (ADI-R)^31^ and Autism Diagnostic Observation Schedule-Generic (ADOS-G)^32^ at age 12 years. The total number of ICD-10 autism symptoms was recorded. The study was approved by the South East Multicentre Research Ethics Committee (REC) (05/MRE01/67). Written informed consent was obtained from all parents.

Of the participants, 48 met consensus criteria for childhood autism and 44 for other pervasive developmental disorders (ICD-10). There were 84 males and 8 females, and the mean age was 15.5 years (s.d. = 0.5, range 14.7–16.8). Mean Wechsler Abbreviated Scale of Intelligence (WASI)^33^ full scale IQ was 84.7 (s.d. = 17.2, range 50–119).

Parental report, when the participants were aged 12, of car ownership and housing tenure were used to construct a crude binary income index (from income differences reported in MacIntryre *et al*^34^) as a family-based measure of deprivation. Neighbourhood deprivation was measured using the Carstairs Index, which combines overcrowding, unemployment, proportion of the population in Registrar General social classes 4 and 5 and households without a car, with total population UK scores ranging from −5.71 (least deprived) to 16.50, median −0.88 (s.d.= 3.41).^35^

### Measures

#### Behavioural and psychiatric measures

**Callous-unemotional (callous–unemotional) traits**. The parent-rated Antisocial Process Screening Device (APSD)^36^ was completed at 16 years to assess characteristics related to psychopathy. The APSD is a 20-item questionnaire with three underlying dimensions of psychopathic behaviour: impulsiveness, narcissism and callous–unemotional traits. The APSD has good internal consistency, reliability and validity^36^ and is predictive of later antisocial outcomes.^37^ The callous–unemotional dimension is made up of six items, scored 0 (not at all true), 1 (sometimes true) or 2 (definitely true): cares about school work, good at keeping promises, feels bad or guilty when does something wrong, concerned about others' feelings, hides feelings from others and keeps the same friends (items are reverse scored where appropriate). For binary classification, adolescents who scored six or above (equal to or above a *T*-score of 65, comparable to the top 6%) on the callous–unemotional subscale were identified as having callous–unemotional traits present.^36^ Internal consistency of the callous–unemotional subscale was moderate in our sample (β = 0.59), similar to that reported elsewhere.^38^ Item response rates are reported in online supplement DS1.

**Behavioural symptoms**. The parent-rated Strengths and Difficulties Questionnaire (SDQ)^39^ at 16 years was used to measure psychiatric symptoms. The SDQ comprises three psychiatric subscales of hyperactivity (ADHD symptoms), conduct and emotional problems, along with further subscales of peer-relationship problems and prosocial behaviour. For binary classification, we used the general population-defined cut-off ≥4 on the conduct subscale for ‘definite’ conduct problems.^39^ Continuous analyses used the conduct, hyperactivity, emotional, peer problems and prosocial subscale scores.

**Autism severity**. Autism severity was measured by dichotomous diagnostic classification of childhood autism/other pervasive developmental disorder (for details see Baird *et al*^28^). In addition, clinicians (G.B., T.C. and E.S.) used the diagnostic information from the ADI-R and ADOS-G to score the 12 ICD-10 symptoms that comprise the ASD diagnoses (score range 0–12, with a higher score indicating greater severity). The parent-rated Social Responsiveness Scale (SRS)^40^ measured the severity of social difficulties associated with ASD. The SRS comprises 65 items providing a total score of autistic traits. This was scored at 12 years in 60 participants and at 16 years in 27, where data were missing at 12 years.

#### Neurocognitive measures

Fuller details of the neurocognitive tasks are given in online supplement DS1. All were administered at 16 years.

**Cognitive ability**. We measured IQ with the WASI^33^ generating full-scale, verbal and performance IQ measures.

**Emotion recognition**. The Ekman–Friesen test of affect recognition was used.^41^ The total number of correct responses for each of the six emotions (happy, sad, fear, surprise, anger, disgust), alongside the total overall score served as the measure of emotion recognition ability. Data from this task have previously been reported by SNAP.^3^

**Theory of mind**. The Strange Stories task^42^ was used as a general measure of mental state understanding. Participants were read a series of stories, which were also available in front of them and accompanied by an appropriate illustration. At the end of each story, they were asked a question about the text. The outcome variable was the average score across the four theory of mind stories (score range 0–2, with a higher score indicating better performance).

The Frith–Happé animations^43^ consist of a series of silent videos of two-dimensional animations, requiring the participant to understand intentionality behind the moving shapes. Four animations depicted theory of mind interactions and two goal-directed interactions. The outcome variable was the average intentionality score for the four theory of mind items (score range 0–5). Data from this task have previously been reported by SNAP.^44^

The Penny Hiding task^45^ was used as a naturalistic and non-verbal measure, specifically indexing the participant's ability to deceive the experimenter. The participant was given six trials of hiding the penny. Responses are coded for the type of deception errors made, with a total score calculated. It was possible to display more than one error on a trial (score range 0–30).

The Combined False Belief task (designed by Rhonda Booth, Institute of Psychiatry, see online supplement DS1 for details of the task) is a combination of first- and second-order false belief tasks based on previous tasks measuring false belief understanding.^46,47^ The two outcome variables were performance on the first- and second-order parts of the story. If the participant failed the false belief question then the overall score was automatically set to zero (score range 0–2 for the first order, 0–3 for the second order false belief task).

The Second-Order False Belief task designed by Bowler was also included, which has greater verbal demands than the combined false belief task.^47^ The outcome variable is whether the participant is able to understand the second-order false belief. Participants were awarded one point for passing the false belief question, and a further point for passing the justification, reality and memory questions; participants failing one or more of these questions were awarded a score of 0 (score range 0–2).

**Executive functioning**. The Card Sort task was used as a measure of cognitive flexibility and response reversal.^48^ Participants had to correctly sort cards to one of three alternative sets across three trials, with the correct set varying in each trial. The key variable was the number of sorts required to reach criterion. In the present analyses, we included only those participants who demonstrated an understanding of the rule in the first trial by reaching criterion before the end. The number of sorts required in the second and third trials was divided into four levels: top half (scores 12–18, *n* = 40), third quartile (scores 19–24, *n* = 21), bottom quartile (scores 25–40, *n* = 17) and those who did not reach criterion by the end of both trials (*n* = 7).

The Trail Making task was included as a measure of attentional switching and response reversal.^49^ Participants were asked to ‘join the dots’ in numerical order, then, in a second trial, in alphabetical order, followed by a third trial switching between numbers and letters. The difference between the time taken on the first and the third trial comprised a measure of switching ability. Because the data were highly skewed, a square-root transformed score was used in the present analyses. Data from this task have previously been reported by SNAP.^50^

### Statistical analysis

All data reduction and statistical analysis were undertaken in Stata version 11. Descriptive statistics are tabulated by group; however, as the group above cut-off for both callous–unemotional traits and conduct problems was small, associations with callous–unemotional traits were explored using the continuous measure. In analysing the relationship among behavioural measures, because of well-recognised cross-domain correlations, any significant bivariate associations were subsequently covaried for conduct problems, in order to identify independent relationships between callous–unemotional traits and behavioural measures. Associations were also covaried for autism severity, to identify associations with callous–unemotional traits above those accounted for by autism severity. The ICD-10 measure was used as a covariate, as it provides an independent measure in addition to parent-rated measures of behaviour. Finally, associations between callous–unemotional traits and performance on the Card Sort and Trail Making tasks were covaried for hyperactivity because of previously reported relationships in other samples. In general, dependent variables were continuous. However, ordinal logistic regression was used for the four-level Card Sort scale. Both the emotion recognition task, in which six different emotions were assessed, and the theory of mind tasks were analysed using multivariate regression to increase efficiency and reduce type 1 errors from multiple testing. For both analyses, the overall combined emotion recognition/theory of mind score was first entered into the regression and subsequently each emotion/theory of mind task was entered as a separate predictor in the regression model. Significance of effects was determined from Wald tests using the robust form of the parameter covariance matrix.

## Results

Of the 92 adolescents with ASD, 51% (95% CI 40–61%, *n* = 47) scored above the designated cut-off for callous–unemotional traits, expected to identify the top 6% of callous–unemotional scores in the general population (equivalent to a *T*-score of 65, according to the APSD manual^36^). Within the high callous–unemotional group (less one participant who was missing SDQ data), only 17% (8/46) also scored above the conduct problems threshold, in contrast to 9% (4/45) with low callous–unemotional scores. These rates of conduct problems were not significantly different (*P* = 0.19, Fisher's exact test).

Table 1 gives the descriptive characteristics for three groups; those below callous–unemotional (CU) and conduct problems (CP) threshold, (CU− CP− group, *n* = 41), those above callous–unemotional but not conduct problems threshold (CU+ CP− group, *n* = 38) and those above threshold for both callous–unemotional and conduct problems (CU+ CP+, *n* = 8). The group with low callous–unemotional and high conduct problems scores was too small to include (*n* = 4). There were no significant differences in age, gender ratio, type of ASD diagnosis or ICD-10 symptom severity among these three groups. The levels of family and neighbourhood disadvantage were also similar. However, consistent with the literature on conduct problems in typically developing adolescents,^51^ the CU+ CP+ group had a significantly lower full-scale and verbal, but not performance IQ.

**Table 1:** Sample characteristics and behavioural symptoms according to the presence of callous–unemotional traits, as rated by the Antisocial Process Screening Device (APSD) and conduct problems, as rated by the Strengths and Difficulties Questionnaire (SDQ)

	CU− CP− (*n* = 41)	CU+ CP− (*n* = 38)	CU+ CP+ (*n* = 8)	Between-group comparisons
Male, *n* (%)	37 (90.2)	35 (92.1)	7 (87.5)	ns
Childhood autism (*v*. other PDD), *n* (%)	20 (48.8)	21 (55.3)	5 (62.5)	ns
ICD-10 symptom severity,^a^ mean (s.d.)	7.78 (2.24)	8.26 (2.64)	9.38 (2.39)	ns
With family disadvantage,^b^ *n* (%)	5 (15.6)	7 (24.1)	1 (16.7)	ns
Neighbourhood deprivation,^c^ mean (s.d.)	−1.09 (2.20)	−0.58 (2.60)	−0.15 (0.81)	ns
WASI, mean (s.d.)				
Full-scale IQ	85.90 (18.08)	85.45 (17.00)	72.00 (12.07)	CU+ CP+ <CU+ CP−, CU− CP−*
Verbal IQ	81.37 (18.05)	83.00 (17.44)	67.50 (10.84)	CU+ CP+ <CU+ CP−, CU− CP−*
Performance IQ	93.20 (19.37)	90.00 (17.55)	81.25 (13.84)	ns
APSD, mean (s.d.)				
Total	10.59 (4.28)	14.55 (4.27)	21.75 (6.18)	CU+ CP+ >CU+ CP− >CU− CP−**
Callous-unemotional	3.63 (1.36)	7.47 (1.29)	8.00 (1.31)	CU+ CP+, CU+ CP− >CU− CP−**
Impulsivity	4.10 (1.77)	4.26 (1.90)	7.13 (2.42)	CU+ CP+, CU+ CP− >CU− CP−**
Narcissism	2.59 (2.26)	2.58 (2.25)	5.50 (3.16)	CU+ CP+, CU+ CP− >CU− CP−**
SQD, mean (s.d.)				
Conduct problems	1.32 (1.04)	1.37 (1.02)	5.25 (1.58)	CU+ CP+ >CU+ CP−, CU− CP−**
Hyperactivity	5.56 (2.76)	5.79 (2.32)	7.25 (2.05)	ns
Emotional symptoms	3.63 (2.55)	3.34 (2.33)	3.75 (2.31)	ns
Peer problems	4.98 (2.38)	5.92 (2.42)	6.25 (2.96)	ns
Prosocial behaviour^d^	6.15 (1.93)	4.22 (2.02)	4.13 (1.89)	CU+ CP+, CU+ CP− <CU− CP−**
SRS total,^e^ mean (s.d.)	83.00 (21.67)	99.57 (20.52)	113.38 (21.00)	CU+ CP+, CU+ CP− >CU− CP−**

APSD, Antisocial Process Screening Device; CP, conduct problems; CU, callous-unemotional traits; ns, non-significant; PDD, pervasive developmental disorder; WASI, Wechsler Abbreviated Scale of Intelligence; SDQ Strengths and Difficulties Questionnaire; SRS Social Responsiveness Scale;

a. CU− CP−, *n* = 37; CU+ CP−, *n* = 34.

b. CU− CP−, *n* = 32; CU+ CP−, *n* = 29; CU+ CP+, *n* = 6.

c. CU− CP−, *n* = 40.

d. CU+ CP−, *n* = 37.

e. CU− CP−, *n* = 39; CU+ CP−, *n* = 37.

* *P*<0.05

** *P*<0.01.

### Behavioural symptoms

Regressions assessing the association between callous–unemotional traits and behavioural symptoms are shown in Table 2. SDQ subscales of conduct problems, hyperactivity and peer-relationship problems were all significantly and positively associated with callous–unemotional traits, whereas prosocial behaviour was negatively related. When conduct problems were accounted for, the associations with peer-relationship problems and prosocial behaviour remained significant but hyperactivity was no longer associated with callous–unemotional traits. Emotional symptoms were unrelated to callous–unemotional traits. The SRS was strongly and significantly associated with callous–unemotional traits and this relationship was unaffected by covarying for conduct problems. When associations were covaried for both conduct problems and autism severity, associations between peer problems and prosocial behaviour and callous–unemotional traits remained significant. Correlations between callous–unemotional traits and psychiatric and autistic symptoms are displayed in online Table DS1.

**Table 2:** Psychiatric and autistic measure associations with callous–unemotional traits as rated by the Antisocial Process Screening Device (APSD) (standardised)^a^

	Unadjusted		Adjusted for conduct problems		Adjusted for conduct problems and autism severity	
Emotional and behavioural problems at 16 (parent-rated)	β	*P*	β	*P*	β	*P*
Strengths and Difficulties Questionnaire						
Conduct problems	0.17	0.02	NA			
Hyperactivity	0.24	0.03	0.18	0.11	0.11	0.38
Emotional symptoms	−0.02	0.82	NA	-	NA	-
Peer relations	0.30	<0.01	0.28	0.01	0.28	0.03
Prosocial behaviour	−0.50	<0.001	−0.51	<0.001	−0.50	<0.001
Social responsiveness scale, total	4.78	<0.001	4.70	<0.001	NA	-

a. Adjusted associations first account for the presence of conduct problems, and second for the presence of conduct problems and level of autism severity as rated by ICD-10 symptom severity.

### Neurocognitive differences among groups

Total emotional recognition and theory of mind task scores by group are shown in Table 3, together with regressions assessing the relationship between callous–unemotional traits and task performance. In line with findings from typically developing populations, there was no overall association between emotion recognition and callous–unemotional traits (*P* = 0.21). As predicted, we identified a specific relationship between higher callous–unemotional traits and poorer recognition of fear (β = −0.30, *P* = 0.02). As we hypothesised *a priori* that fear recognition would be selectively affected, no corrections for multiple comparisons were performed. When full-scale IQ was controlled for, the relationship between callous–unemotional traits and fear recognition remained significant (β = −0.24, *P* = 0.04). No overall association was found between callous–unemotional traits and either overall theory of mind (*P* = 0.73) or with any individual theory of mind measure.

**Table 3:** Neurocognitive correlates of callous–unemotional traits as rated by the Antisocial Process Screening Device (APSD): scores and multivariate regression of callous-unemotional traits on emotional recognition and theory of mind tasks (standardised)

	Group, mean (s.d.)			Unadjusted test statistic			Group, *n*		
	CU− CP−	CU+ CP−	CU+ CP+	*F* (d.f.)	β	*P*	CU− CP−	CU+ CP−	CU+ CP+
*Emotion recognition*									
Overall test of effect				1.44 (6,87)		0.21	40	36	8
Specific emotion							40	36	8
Happiness	9.88 (0.40)	9.94 (0.23)	9.38 (1.41)		−0.01	0.71			
Sadness	7.55 (2.37)	7.14 (1.94)	7.13 (0.64)		−0.15	0.13			
Fear	6.35 (2.65)	5.50 (2.85)	5.38 (2.00)		−0.30	0.02			
Anger	7.18 (1.93)	6.75 (1.87)	7.50 (1.93)		−0.03	0.76			
Surprise	8.30 (2.57)	8.50 (1.98)	7.50 (2.73)		−0.11	0.32			
Disgust	4.85 (3.04)	4.53 (2.97)	3.63 (1.77)		−0.20	0.12			
*Theory of mind*									
Overall test of effect				0.63 (7,76)		0.73	41	38	8
Specific task									
Strange Stories	0.76 (0.49)	0.98 (0.54)	0.50 (0.32)		0.01	0.86	37	34	7
Penny Hiding Errors	1.66 (0.73)	1.84 (0.82)	1.75 (0.89)		0.02	0.64	41	38	8
Frith-Happé animations	2.91 (1.07)	2.86 (0.87)	2.67 (0.86)		−0.03	0.48	38	34	6
Combined First Order False Belief	1.71 (0.68)	1.68 (0.71)	1.50 (0.93)		−0.00	0.86	41	37	8
Combined Second Order False Belief	1.71 (1.12)	1.59 (1.19)	1.63 (1.19)		−0.03	0.53	41	37	8
Second Order False Belief	1.61 (0.93)	1.67 (0.78)	1.57 (0.98)		−0.03	0.44	36	33	7

CP, conduct problems; CU, callous–unemotional traits.

There was no association between callous–unemotional traits and cognitive flexibility as measured using the Card Sort (odds ratio (OR) = 1.06, *P* = 0.44) or difference scores on the Trail Making task (β = 0.11, *P* = 0.30). Controlling for hyperactivity left the pattern of results unchanged. Total scores by group are displayed in Table 4. Correlations between callous–unemotional traits and all neurocognitive measures are displayed in online Tables DS2 and DS3.

**Table 4:** Neurocognitive correlates of callous–unemotional traits as rated by the Antisocial Process Screening Device (APSD): scores and association with callous–unemotional traits on Trail Making and Card Sort tasks (standardised)

	CU− CP− group (*n* = 41)	CU+ CP− group (*n* = 36)	CU+ CP+ group (*n* = 8)	β	Odds ratio	*P*
Trail making difference score, mean (s.d.)	7.22 (2.23)	7.21 (2.63)	7.21 (1.84)	0.11		0.30
Card sort trials to criterion, *n*					1.06	0.44
Top 50%	20	16	4			
51–75%	9	12	0			
76%+	7	6	4			
Did not meet criterion	5	2	0			

CP, Conduct problems; CU, Callous–unemotional traits.

## Discussion

To our knowledge, this is the first report of the prevalence, psychiatric correlates and neurocognitive profile of callous–unemotional traits within adolescents with ASD using a population-based sample. We found that 51% scored in the top 6% for callous–unemotional traits, reflecting nearly a tenfold increase over the general population.^52,53^ Our prevalence rate is higher than that reported in a previous study of callous–unemotional traits in a smaller, selected sample of young people with ASD, which found 36% were ‘high’ in callous–unemotional traits.^26^ The previous study used teacher-reported callous–unemotional traits and ours parent-report; informant type could be related to these differences. Both previous work and our results suggest an increase in callous–unemotional traits in individuals with ASD, compared with the general population. We also demonstrated that callous–unemotional traits in adolescents with ASD are associated with the same deficit in fear recognition previously reported in typically developing samples.

### Causes of the high prevalence of callous–unemotional traits in ASD

There are two possible explanations for the remarkably high prevalence of callous–unemotional traits in our sample of adolescents with ASD. First, people with ASD may be at increased risk of developing callous–unemotional traits. It may be that the cognitive impairments associated with ASD increase the risk of developing these traits. Second, the overlap could be as a result of superficial similarity in behaviours, assessed on measures such as the APSD, which may be in part tapping ASD characteristics, leading to an artificial inflation of estimates of callous–unemotional traits. Questionnaires measuring characteristics such as empathy may fail to distinguish affective and cognitive components, which some argue are distinct traits, and are differentially associated with ASD and callous–unemotional traits.^22–24^ As some behavioural characteristics associated with callous–unemotional traits can be superficially similar to features of ASD (such as lack of sensitivity to the feelings of others), instruments designed to measure callous–unemotional traits in typical populations may not be sensitive enough to discriminate between overlapping aspects of ASD and callous–unemotional traits (for example being able to identify *v.* caring about emotions of others). However, as we found the specific deficit in fear recognition difficulty associated with callous–unemotional traits in non-ASD populations, we suggest that the APSD may be sensitive enough to distinguish a meaningfully different subgroup of individuals with ASD with additional callous–unemotional traits. It would be interesting in future work to examine alexithymia (impaired ability to reflect on and report own emotions) in this subgroup, given recent evidence that emotion processing and empathy deficits are a function of co-occurring alexithymia rather than characteristics of ASD itself.^54^

Within our total sample of adolescents with ASD, 13% (12/92) scored above a cut-off indicative of conduct problems. This rate is similar to those reported in samples of typically developing adolescents.^51^ Of the group classified as above cut-off for callous–unemotional traits, only 17% displayed concurrent conduct problems. This contrasts with general population findings; in a community sample of 7- to 12-year-olds, those with callous–unemotional traits showed a 0.95 probability of having concomitant conduct problems, and only 0.2% of those with these traits scored below cut-off for conduct problems.^52^ The dissociation of callous–unemotional from conduct problems in ASD may indicate a different cognitive underpinning. Alternatively, the sociocognitive difficulties associated with ASD may lead to reduced exposure to peers and prevent individuals from engaging in the more socialised and peer-related aspects of conduct-disordered behaviour.

### Behavioural correlates of callous–unemotional traits in ASD

Callous–unemotional traits were associated with an increase in conduct problems, hyperactivity and peer problems, and a decrease in prosocial behaviour. However, when conduct problems were accounted for, the relationship between callous–unemotional traits and hyperactivity became non-significant. Given the conceptualisation of callous–unemotional traits as being in part because of an inability to emotionally resonate with others, our finding of increased callous–unemotional traits being associated with difficulties in interpersonal functioning (as indexed by increased peer problems and a decrease in prosocial behaviour) was expected, and is in line with previous research.^13,51^ Our results therefore provide some evidence for similar behavioural profiles associated with callous–unemotional traits in individuals with ASD, as in those without ASD.

Surface-level similarities in callous–unemotional traits and ASD with respect to impairments in interpersonal functioning may lead to certain behavioural problems being misinterpreted as being solely because of ASD, whereas callous–unemotional traits may also be driving this behaviour. Understanding of the behavioural correlates of these traits in ASD may be vital to unpicking the heterogeneous presentation of ASD. Although the association of callous–unemotional traits with prosocial behaviour and peer problems could be simply manifestations of more severe ASD, we suggest this is not the case. When both conduct problems and autism severity were accounted for, the relationships between callous–unemotional traits and both prosocial behaviour and peer problems remained, suggesting these traits selectively have an impact on the presentation of ASD beyond difficulties driven by autism severity.

### Neurocognitive profile of callous–unemotional traits in ASD

The present findings partially support the idea that the cognitive correlates of callous–unemotional traits in people with ASD are similar to those in the typically developing population, suggesting a shared underlying aetiology. Those with callous–unemotional traits and concurrent conduct problems had significantly lower verbal and full-scale IQ than both those with callous–unemotional traits alone and those scoring below threshold for both callous–unemotional traits and conduct problems. This association is in line with previous research.^51^ No differences were found between individuals scoring below the callous–unemotional and conduct problems threshold, or above the threshold for callous–unemotional traits without conduct problems, suggesting the IQ relationship is with conduct problems.

Previous literature also reports difficulties in the recognition of fearful and sad emotions in children and adolescents with psychopathic and callous–unemotional traits^14,15^ and individuals with ASD with a history of offending.^27^ In line with our predictions, our findings of a similar selective association between callous–unemotional traits and fear recognition in adolescents with ASD supports the idea of comparable disturbance in affective domains, and subsequently similar aetiology of callous–unemotional traits in individuals with and without ASD. Unlike previous studies of callous–unemotional traits in individuals with ASD,^27^ we did not find impairment in the recognition of sad faces; this may have been because of decreased power within our sample. We found no differences on any of the theory of mind tasks, suggesting no additional impairment in cognitive domains associated with callous–unemotional traits among individuals with ASD, similar to previous findings.^22,23^ The lack of association between callous–unemotional traits and theory of mind is unlikely to be because of theory of mind task insensitivity, as all tasks aside from the Second Order False Belief task showed a significant relationship with at least one measure of autism severity (online Table DS4). Our findings support a selective affective difficulty but no sociocognitive difficulties, similar to that reported in individuals with callous–unemotional traits without ASD.^22–24^

We found no association between callous–unemotional traits and cognitive flexibility, consistent with previous findings in ASD^22,23^ but different from those in the general population.^18^ Previous studies suggest associations between callous–unemotional traits and measures of executive functioning may be driven by comorbid ADHD,^20^ thus we accounted for hyperactivity within our analyses. This did not change our results, suggesting callous–unemotional traits may indeed present differently with respect to cognitive flexibility when found in individuals with ASD, however these findings require replication.

### Strengths and limitations

Strengths of this study include use of a population-based sample of adolescents with carefully characterised ASD. A wide range of psychiatric and cognitive characteristics were assessed, including many that have been previously related to callous–unemotional traits in the typically developing population. This allowed us to explore a range of variables involved in the presentation of callous–unemotional traits, and the sample used suggests any conclusions drawn regarding prevalence rates of callous–unemotional traits in ASD and their psychiatric/cognitive correlates are likely to be representative of the adolescent ASD population.

Limitations of the present study include the use of mainly parent-reported questionnaires of behavioural symptoms, including callous–unemotional traits; however the validity of self-reported behavioural measures in the ASD population still needs to be established. Behavioural items indexing callous–unemotional traits on the APSD may overlap with ASD symptoms, thus more sensitive measures of these traits in ASD require development. Second, we did not have sufficient statistical power to include a comparison group of typically developing adolescents with which to compare our findings. Ideally, future work would examine callous–unemotional traits in typically developing samples compared with individuals with ASD matched for intellectual level.

### Implications

Clinicians have been concerned that some people with ASD appear to show high levels of callous–unemotional traits and the present study supports this clinical impression for a subset of individuals. However, callous–unemotional traits in individuals with ASD are less strongly associated with conduct problems than is the case in individuals without ASD. Therefore clinicians should be sensitive to the differential behavioural correlates associated with callous–unemotional traits in individuals with ASD, as compared with other clinical groups. Parents, caregivers and other people working with individuals with ASD may require specific psychoeducation about these traits, which are often distressing to others but are not necessarily a predictor of antisocial behaviour.

The present findings partially support a shared aetiology of callous–unemotional traits in people with ASD and the general population as indexed by impairments in affective domains, but also suggest differences, particularly in the realm of cognitive flexibility. Further study of callous–unemotional traits from both a behavioural and cognitive perspective in individuals with ASD with and without conduct problems would clarify the role of these traits in predisposing individuals to conduct problems and antisocial behaviour.
